# Life After Traumatic Brain Injury: Effects on the Lifestyle and Quality of Life of Community-Dwelling Patients

**DOI:** 10.1089/neur.2023.0113

**Published:** 2024-03-01

**Authors:** Yi-Chia Wei, Chih-Ken Chen, Chemin Lin, Yu-Chiau Shyu, Pin-Yuan Chen

**Affiliations:** ^1^ Department of Neurology, Chang Gung Memorial Hospital, Keelung Branch, Keelung, Taiwan.; ^2^ College of Medicine, Chang Gung University, Taoyuan, Taiwan.; ^3^ Community Medicine Research Center, Chang Gung Memorial Hospital, Keelung Branch, Keelung, Taiwan.; ^4^ Department of Psychiatry, Chang Gung Memorial Hospital, Keelung Branch, Keelung, Taiwan.; ^5^ Department of Nursing, Chang Gung University of Science and Technology, Taoyuan, Taiwan.; ^6^ Department of Neurosurgery, Chang Gung Memorial Hospital, Keelung Branch, Keelung, Taiwan.

**Keywords:** analgesics, anxiety, community cohort, nutrition, quality of life, sarcopenia, traumatic brain injury

## Abstract

Persons who have experienced traumatic brain injury (TBI) may encounter a range of changes in their physical, mental, and cognitive functions as well as high fatigue levels. To gain a comprehensive understanding of the challenges faced by persons after TBI, we conducted multi-domain assessments among community-dwelling persons with a history of TBI and compared them with age- and sex-matched controls from the Northeastern Taiwan Community Medicine Research Cohort between 2019 and 2021. A total of 168 persons with TBI and 672 non-TBI controls were not different in terms of demographics, comorbidities, and physiological features. However, compared with the non-TBI group, the TBI group had a distinct lifestyle that involved increased reliance on analgesics (6.9% vs. 15.0%, respectively; *p* = 0.001) and sleep aids (*p* = 0.008), which negatively affected their quality of life. Moreover, they consumed more coffee (*p* < 0.001), tea (*p* < 0.001), cigarettes (*p* = 0.002), and betel nuts (*p* = 0.032) than did the non-TBI group. Notably, the use of coffee had a positive effect on the quality of life of the TBI group (*F* = 4.034; *p* = 0.045). Further, compared with the non-TBI group, the TBI group had increased risks of sarcopenia (*p* = 0.003), malnutrition (*p* = 0.003), and anxiety (*p* = 0.029) and reduced blood levels of vitamin D (29.83 ± 10.39 vs. 24.20 ± 6.59 ng/mL, respectively; *p* < 0.001). Overall, the TBI group had a reduced health-related quality of life, with significant challenges related to physical health, mental well-being, social interactions, pain management, and fatigue levels. Moreover, the TBI group experienced poorer sleep quality and efficiency than did the non-TBI group. In conclusion, persons who have sustained brain injuries that require comprehensive and holistic care that includes lifestyle modification, mental and physical healthcare plans, and increased long-term support from their communities.

ClinicalTrials.gov (identifier: NCT04839796)

## Introduction

Traumatic brain injury (TBI) is characterized by changes in brain function or structure attributable to an external force. TBI can manifest as different degrees of severity.^1^ It is a common condition that affects the daily lives of persons and may lead to a sequela of brain injury. The age-standardized incidence of TBI is 369 per 100,000 persons.^2^ Globally, the number of TBI survivors is approximately 55.5 million; this high number imposes a substantial healthcare burden on both society and survivors.^2^ A diverse group of persons with various roles is required to ensure adequate healthcare for patients with TBI. This group comprises TBI patients, their families, healthcare professionals specializing in TBI care, and the community. Various factors, such as mental, physical, psychosocial, educational, and medical factors as well as community integration, must be considered for TBI healthcare.^3^ Moreover, various quality indicators and outcome domains must be considered for a comprehensive assessment of persons with TBI.^4^

In this study, we assessed various health and lifestyle parameters of community-dwelling persons who have experienced TBI. Our approach involved identifying patients from the Northern coastal Taiwan community cohort and subsequently conducting comprehensive multi-domain assessments. The results were compared between patients and matched controls from the same cohort. Our objective was to establish the foundation for future interventions aimed at enhancing the quality of life of community-dwelling persons with a history of TBI.

## Methods

### Study cohort

Persons with a history of TBI were identified from the Northeastern Taiwan Community Medicine Research Cohort (NTCMRC; ClinicalTrials.gov identifier: NCT04839796) between 2019 and 2021. The NTCMRC was conducted by the Community Medicine Research Center of the Keelung Chang Gung Memorial Hospital. The Institutional Review Board of the Chang Gung Memorial Hospital reviewed and approved the research protocol (approval nos.: 201901351B0 and 201800289A3). Before their inclusion in the study, all participants provided written consent.

**FIG. 1.**
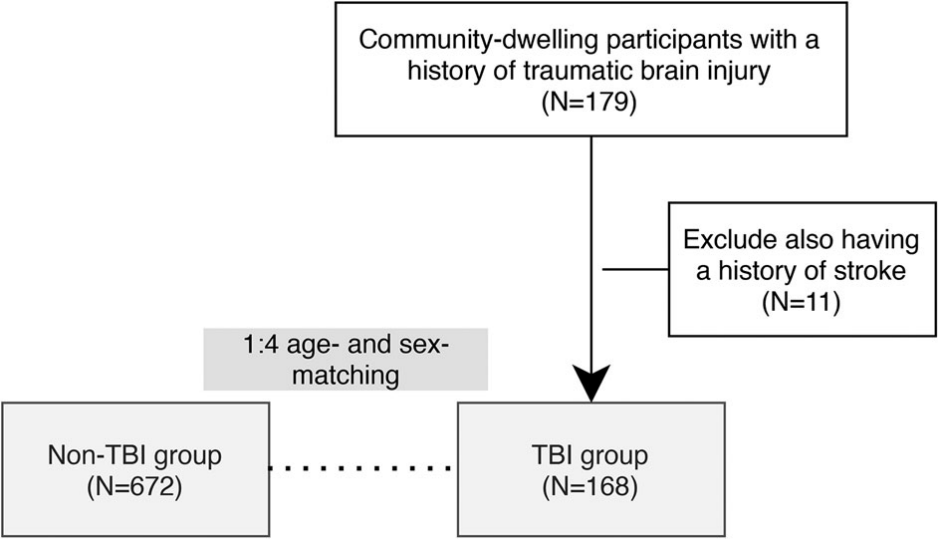
Participant enrollment. Participants of the Northeastern Taiwan Community Medicine Research Cohort (NTCMRC) were queried for a history of TBI. Among 179 participants who reported brain trauma history, 11 were excluded for also having a stroke history. The final enrollment of 168 TBI participants were matched with age and sex to set a control group (non-TBI). TBI, traumatic brain injury.

Community-dwelling persons with TBI were enrolled on the basis of their responses to the question “Have you ever hit your head in a fall, car accident, or trauma?” and “Did you lose consciousness at that time?” A total of 179 persons reported positive brain trauma events; of them, 11 were excluded because of a history of stroke. Finally, 168 persons with a history of TBI (TBI group) were included in the analysis. For comparison, we included age- and sex-matched (1:4) persons without TBI (non-TBI group; Fig. 1).

### Outcome measures

The primary outcome was independence in the performance of activities of daily life, which indicated total self-care by the participants. By contrast, dependency encompassed partial self-care and total dependence on others' care.

The secondary outcome was the quality of daily life, which was assessed using the RAND-36 scoring system. This system was derived from a 36-item short-form survey^5,6^ and had been validated in the Chinese version.^7^ The RAND-36 score was calculated as the sum of scores (RAND-36_total) on the following eight domains: physical functioning, role limitations attributable to physical functioning, role limitations attributable to emotional functioning, energy/fatigue, emotional well-being, social functioning, pain score, and general health perceptions. Higher scores on the RAND-36 scale indicated a better quality of life.

To assess the participants' sleep quality, we used the Chinese version of the Pittsburgh Sleep Quality Index (PSQI).^8^ This tool comprises 19 items that are used to measure seven aspects of sleep quality. The response on each item is rated on a scale with end-points ranging from 0 to 3. The sum of scores on these seven components yields a global score, which ranges from 0 to 21 and represents overall sleep quality. Higher global scores indicate poorer sleep quality.^8,9^

### Demographics, lifestyle, and physiological features

We collected participants' demographic data, including age, sex, education level, and marital status. Additionally, data on the regular consumption of alcohol, betel nut, coffee, and tea as well as the habit of smoking were obtained. Level of alcohol use was determined using the Chinese version of the Alcohol Use Disorders Identification Test (https://auditscreen.org), which has been validated in Taiwan.^10^ This test comprises 10 questions. The total score ranges from 0 to 40; a higher score indicates a higher risk of alcohol abuse.^10,11^ Participants were also asked about their current medications, which were categorized as follows: antihypertensive drugs, lipid-lowering drugs, hypoglycemic drugs, analgesics, and hormone-containing medicines. Further, participants' body height, weight, body mass index, blood pressure, and heart rate were recorded.

### Definition of medical conditions

Diabetes mellitus was diagnosed on the basis of the following criteria: a fasting blood sugar level of ≥126 mg/dL, a glycated hemoglobin percentage of ≥6.5, the use of oral hypoglycemia or insulin drugs, or a medical history of diabetes mellitus.

Metabolic syndrome (MetS) was defined as either an MetS score of ≥3 or the use of an ordinal scoring approach.^12^ It was determined on the basis of the guidelines of Taiwan's Health Promotion Administration, Ministry of Health and Welfare.^13^ The diagnostic criteria for MetS were as follows: waist circumference of ≥90 cm (35 inches) for men and ≥80 cm (31 inches) for women, systolic blood pressure of ≥130 mm Hg or diastolic blood pressure of ≥85 mm Hg or the use of prescribed medication for hypertension, fasting blood glucose level of ≥100 mg/dL or the use of doctor-prescribed medications for diabetes management, fasting triglyceride level of ≥150 mg/dL or the use of doctor-prescribed triglyceride-reducing medications, and high-density lipoprotein cholesterol level of <40 mg/dL for men and <50 mg/dL for women. Persons meeting three or more of the five aforementioned criteria were diagnosed as having MetS.

Chronic kidney disease was defined as an estimated glomerular filtration rate of <60 mL/min/1.73 m^2^, overt proteinuria with a urine protein level of 1+, an albumin-to-creatinine ratio of ≥30 mg/g, or a urine protein-to-creatine ratio of ≥150 mg/g.^14^

### Laboratory data

Peripheral blood samples collected from participants were subjected to a battery of tests assessing renal function and measuring the levels of fasting glucose and glycated hemoglobin (glucose metabolism profile), liver enzyme, high-sensitivity c-reactive protein, albumin, total protein, and uric acid. Additionally, the lipid profile was analyzed; for this, we measured the levels of low-density lipoprotein, high-density lipoprotein, triglyceride, hemoglobin, hematocrit, and vitamin D. Laboratory tests were conducted at Chang Gung Memorial Hospital, Keelung.

### Study tools

#### Strength, Assistance in walking, Rise from a chair, Climb stairs, and Falls questionnaire

The SARC-F (Strength, Assistance in walking, Rise from a chair, Climb stairs, and Falls) questionnaire is a straightforward screening tool used for the detection of sarcopenia among older persons. This questionnaire has five components: strength, assistance with walking, rising from a chair, climbing stairs, and falls. The total score ranges from 0 to 10; impairments in any of the five components contribute to the total score. Specifically, impairment in each of the aforementioned aspects of sarcopenia adds 0–2 points to the total score. A SARC-F score of ≥4 indicates the presence of sarcopenia.^15^ The Chinese version of SARC-F, which has been used, has been validated among the Taiwanese and Chinese populations.^16,17^

#### Mini Nutritional Assessment

The Mini Nutritional Assessment (MNA) instrument, a validated nutrition screening tool developed in 1989, is used to assess the nutritional status of older persons,^18^ and validated in the traditional Chinese version.^19^ It comprises 18 questions; the total score ranges from 0 to 30. A score of <24 indicates a potential risk of malnutrition, whereas a score of <17 points suggests malnourishment.^19^

#### Hospital Anxiety and Depression Scale

The Hospital Anxiety and Depression Scale (HADS) was used to evaluate the participants' susceptibility to depression and anxiety. This scale comprises seven questions aimed at evaluating depression and anxiety. The participants' mental state was appraised using two subscales: the HADS anxiety and depression subscales. The total scores on these subscales range from 0 to 21; a higher score indicates a higher severity of anxiety or depression.^20^ We used the traditional Chinese version of HADS, which had been validated in Taiwan.^21^

#### Short-Form UCLA Loneliness Scale

The self-reported UCLA Loneliness Scale comprises 20 items.^22^ However, a validated shorter version in Chinese (comprising only eight items) of this scale is available for reliably measuring loneliness.^23^ The total score on this shorter version ranges from 8 to 32; higher scores indicate higher levels of loneliness.^23,24^

#### Ascertain Dementia 8

The Ascertain Dementia 8 (AD8) questionnaire was used to investigate cognitive disabilities among our participants. This questionnaire comprises eight concise questions focusing on various areas, such as orientation, judgment, finance management, appointment remembering, appliances, interest, repeats, and consistent cognitive changes.^25^ The traditional Chinese version of this questionnaire has been validated in a Taiwanese study.^26^

### Statistical analysis

The independent-samples *t*-test was used for the between-group comparisons of continuous data. Categorical data were compared using the chi-square test. In addition, the interaction between the TBI and non-TBI groups was analyzed. The effects of TBI and analgesic use on the participants' quality of life were analyzed using the two-way analysis of variance test. A *p* value of <0.05 was considered statistically significant.

## Results

### Participant demographics

As shown in Table 1, no significant difference was found between the TBI and non-TBI groups in age (58.11 ± 13.30 vs. 58.04 ± 13.23, respectively; *p* = 0.954), sex (62.5% vs. 62.4% female, respectively; *p* = 0.972), education level (*p* = 0.094), or marital status (*p* = 0.545).

**Table 1.** Demographics

	TBI (*N* = 168)	Non-TBI (*N* = 672)	*p *value
Age	58.11 ± 13.30	58.04 ± 13.23	0.954
Sex (female)	105 (62.5%)	419 (62.4%)	0.972
Education			0.094
Cannot read	9 (5.4%)	32 (10.5%)	
Elementary	28 (16.9%)	67 (21.9%)	
Junior high	28 (16.9%)	46 (15.0%)	
High school	55 (33.1%)	82 (26.8%)	
Collage	37 (22.3%)	72 (23.5%)	
Post-graduate	9 (5.4%)	7 (2.3%)	
Marriage			0.545
Not married	23 (13.7%)	30 (10.1%)	
Married	127 (75.6%)	238 (79.9%)	
Divorced	8 (4.8%)	10 (3.4%)	
Widowed	10 (6.0%)	20 (6.7%)	

TBI, traumatic brain injury.

### Lifestyle and comorbidities

Compared with the non-TBI group, TBI group had increased proportions of participants who smoked (22.2% vs. 33.9%, respectively; *p* = 0.002) and chewed betel nuts (6.9% vs. 11.9%, respectively; *p* = 0.032). Additionally, a higher proportion of persons in the TBI group than in the non-TBI group reported the consumption of coffee (62.5% and 26.4%, respectively; *p* < 0.001) and tea (49.4% vs. 28.2%, respectively; *p* < 0.001).

Regarding comorbidities, no significant difference was noted between the TBI and non-TBI groups in terms of MetS (28.0% vs. 29.6%, respectively; *p* = 0.677) and the distribution of MetS-related scores (*p* = 0.325). Similarly, no notable variations were observed between the TBI and non-TBI groups in terms of chronic kidney disease (19.0% vs. 18.5%, respectively; *p* = 0.859); diabetes mellitus (13.1% vs. 17.9%, respectively; *p* = 0.141); or current use of oral hypoglycemic agents or insulin (9.5% vs. 11.8%, respectively; *p* = 0.415), antihypertensive drugs (23.2% vs. 29.0%, respectively; *p* = 0.281), lipid-lowering drugs (13.7% vs. 12.5%, respectively; *p* = 0.682), or hormone drugs (2.4% vs. 2.1%, respectively; *p* = 0.824). However, the proportion of persons using analgesics for managing pain problems was higher in the TBI group than in the non-TBI group (15.0% vs. 6.9%, respectively; *p* = 0.001). The overall between-group differences suggested that the demand for cigarettes, betel nuts, coffee, tea, and physical pain management was higher in the TBI group than in the non-TBI group (Table 2).

**Table 2.** Lifestyles and Comorbidities

	TBI (*N* = 168)	Non-TBI (*N* = 672)	*p *value
Smoking	57 (33.9%)	148 (22.2%)	0.002^*^
Betel nut chewing	20 (11.9%)	46 (6.9%)	0.032^*^
Coffee	105 (62.5%)	176 (26.4%)	<0.001^*^
Tea	83 (49.4 %)	188 (28.2%)	<0.001^*^
AUDIT	2.38 ± 3.48	2.36 ± 4.38	0.960
Metabolic syndrome	47 (28.0%)	199 (29.6%)	0.677
Metabolic syndrome score			0.325
0 (low risk)	43 (25.6%)	154 (22.9%)	
1	40 (23.8%)	164 (24.4%)	
2	38 (22.6%)	155 (23.1%)	
3	34 (20.2%)	113 (16.8%)	
4	11 (6.5%)	55 (8.2%)	
5 (high risk)	2 (1.2%)	31 (4.6%)	
Heart failure	0 (0.0%)	26 (3.9%)	0.010^*^
Chronic kidney disease	32 (19.0%)	124 (18.5%)	0.859
Diabetes mellitus	22 (13.1%)	120 (17.9%)	0.141
Using OHA/insulin	16 (9.5%)	77 (11.8%)	0.415
Using antihypertensive drugs	39 (23.2%)	195 (29.0%)	0.281
Using lipid-lowering drugs	23 (13.7%)	76 (12.5%)	0.682
Using hormone drug	4 (2.4%)	14 (2.1%)	0.824
Using analgesics	25 (15.0%)	45 (6.9%)	0.001^*^

AUDIT, Alcohol Use Disorders Identification Test; OHA, oral hypoglycemic agent; TBI, traumatic brain injury.

### Physiological and laboratory features

No notable between-group differences were found in body configurations (e.g., weight, waist circumference, and body mass index) or physiological markers (e.g., blood pressure and heart rate; Table 3).

**Table 3.** Physiological Features

	TBI (*N* = 168)	Non-TBI (*N* = 672)	*p *value
Weight (kg)	63.24 ± 13.51	63.88 ± 12.47	0.557
Waist (cm)	81.97 ± 11.57	82.63 ± 10.53	0.477
BMI	24.87 ± 4.47	25.11 ± 4.00	0.491
SBP (mm Hg)	128.36 ± 17.30	129.95 ± 19.90	0.341
DBP (mm Hg)	76.40 ± 9.74	77.63 ± 12.48	0.169
HR (per min)	78.98 ± 11.42	78.22 ± 11.83	0.454

BMI, body mass index; SBP, systolic blood pressure; DBP, diastolic blood pressure; HR, heart rate; TBI, traumatic brain injury.

Blood analyses revealed that compared with the non-TBI group, the TBI group had a normal but slightly low estimated glomerular filtration rate (90.44 ± 23.28 vs. 85.83 ± 20.05 mL/min/1.73 m^2^, respectively; *p* = 0.010) and slightly high liver enzyme alanine transaminase (24.63 ± 13.95 vs. 28.48 ± 16.60 U/L, respectively; *p* = 0.006). Significant between-group differences were observed in the blood level of vitamin D (TBI vs. non-TBI groups: 24.20 ± 6.59 vs. 29.83 ± 10.39 ng/mL, respectively; *p* < 0.001), suggesting a higher risk of vitamin D deficiency in the TBI group than in the non-TBI group. Moreover, no between-group differences were noted in lipid profile, blood glucose profile, or the levels of hemoglobin, hematocrit, high-sensitivity c-reactive protein, uric acid, and albumin (Table 4).

**Table 4.** Laboratory Tests

	TBI (*N* = 168)	Non-TBI (*N* = 672)	*p *value
Creatinine (mg/dL)	0.83 ± 0.32	0.79 ± 0.26	0.103
eGFR (mL/min/1.73 m^2^)	85.83 ± 20.05	90.44 ± 23.28	0.010^*^
ALT (U/L)	28.48 ± 16.60	24.63 ± 13.95	0.006^*^
AST (U/L)	24.63 ± 9.85	24.00 ± 10.33	0.482
Bilirubin total (mg/dL)	0.60 ± 0.29	0.72 ± 0.32	<0.001^*^
hs-CRP (mg/L)	2.01 ± 2.57	2.18 ± 3.42	0.572
Albumin (g/dL)	4.66 ± 0.30	4.67 ± 0.28	0.474
Total protein (g/dL)	7.40 ± 0.45	7.38 ± 0.42	0.619
Uric acid (mg/dL)	5.63 ± 1.30	5.62 ± 1.46	0.934
Triglyceride (mg/dL)	129.73 ± 83.47	125.07 ± 92.82	0.554
Cholesterol total (mg/dL)	198.41 ± 39.17	197.70 ± 39.87	0.836
LDL (mg/dL)	124.94 ± 35.83	122.18 ± 34.69	0.360
VLDL (mg/dL)	25.46 ± 14.44	24.55 ± 15.45	0.488
Non-HDL (mg/dL)	140.98 ± 37.44	139.89 ± 38.89	0.744
HDL (mg/dL)	57.43 ± 16.48	57.79 ± 15.92	0.799
T-Chol/HDL (%)	3.68 ± 1.11	3.64 ± 1.13	0.660
LDL/HDL (%)	2.33 ± 0.91	2.26 ± 0.85	0.307
HbA1c (%)	5.77 ± 0.75	5.90 ± 0.81	0.058
HOMA-IR	2.71 ± 1.98	2.71 ± 3.58	0.993
Fasting glucose (mg/dL)	99.58 ± 18.47	102.53 ± 27.44	0.187
Hemoglobin (g/dL)	13.88 ± 1.48	13.75 ± 1.58	0.360
Hematocrit (%)	41.45 ± 3.91	41.04 ± 4.05	0.237
Vitamin D (ng/mL)	24.20 ± 6.59	29.83 ± 10.39	<0.001^*^

eGFR, estimated glomerular filtration rate; ALT, alanine transaminase; AST, aspartate transaminase; hs-CRP, high-sensitivity c-reactive protein; LDL, low-density lipoprotein; VLDL, very low-density lipoprotein; HDL, high-density lipoprotein; HbA1c, glycated hemoglobin; HOMA-IR, homeostatic model assessment for insulin resistance; TBI, traumatic brain injury.

### Multi-domain questionnaire assessments

A total of 168 participants with TBI and 55 non-TBI controls completed questionnaires regarding sarcopenia, nutrition, anxiety, depression, loneliness, and subjective cognition. Compared with non-TBI participants, the TBI group exhibited an elevated risk of sarcopenia (SARC-F: 1.01 ± 1.50 vs. 0.47 ± 1.02, respectively; *p* = 0.003), a slightly reduced state of nutrition (not reaching the point of malnutrition; MNA: 25.21 ± 3.37 vs. 26.43 ± 2.27, respectively; *p* = 0.003), and an increased degree of anxiety (HADS anxiety: 5.23 ± 4.19 vs. 3.83 ± 3.42, respectively; *p* = 0.029). However, no between-group difference in the degree of depression, loneliness, or subjective cognitive complaints was observed (all *p* > 0.05; Table 5).

**Table 5.** Multi-Domain Questionnaire Assessments

	TBI (*N* = 168)	Non-TBI (*N* = 55)	*p *value
SARC-F	1.01 ± 1.50	0.47 ± 1.02	0.003^*^
MNA	25.21 ± 3.37	26.43 ± 2.27	0.003^*^
HADS-A	5.23 ± 4.19	3.83 ± 3.42	0.029^*^
HADS-D	4.70 ± 3.79	4.67 ± 3.61	0.952
ULS-8	15.02 ± 5.25	14.69 ± 4.64	0.672
AD8	2.00 ± 2.48	1.45 ± 2.17	0.146

SARC-F, Strength, Assistance with walking, Rise from a chair, Climb stairs, and Falls; MNA, Mini Nutritional Assessment; HADS, Hospital Anxiety and Depression Scale; ULS-8, short-form UCLA Loneliness Scale; AD8, Ascertain Dementia 8; TBI, traumatic brain injury.

### Quality of life and independence

No significant difference was observed between the TBI and non-TBI groups in the proportion of participants who were dependent on others for the activities of daily life (3.0% vs. 2.5%, respectively; *p* = 0.193) or that of participants who were capable of self-care (97.0% vs. 98.5%, respectively; *p* = 0.350). The RAND-36 test was used to evaluate the health-related quality of life of the participants with or without TBI. The results revealed that the TBI group had lower scores in all eight domains than did the non-TBI group (Table 6): physical functioning (82.33 ± 20.96 vs. 87.12 ± 18.37, respectively; *p* = 0.004), role limitations attributable to physical health (72.22 ± 39.80 vs. 82.68 ± 34.78, respectively; *p* = 0.002), role limitations attributable to emotional problems (76.71 ± 38.75 vs. 87.72 ± 30.41, respectively; *p* = 0.001), energy and fatigue (60.74 ± 20.15 vs. 69.13 ± 19.71, *p* < 0.001), emotional well-being (65.57 ± 18.04 vs. 73.69 ± 17.50, respectively; *p* < 0.001), social functioning (82.45 ± 18.82 vs. 90.19 ± 15.15, respectively; *p* < 0.001), pain (78.04 ± 19.49 vs. 84.06 ± 17.93, respectively; *p* < 0.001), and general health (57.04 ± 21.47 vs. 63.77 ± 19.77, respectively; *p* < 0.001).

**Table 6.** Outcome Comparison

	TBI (*N* = 168)	Non-TBI (*N* = 672)	*p *value
Dependent	5 (3.0%)	10 (2.5%)	0.193
Self-care			0.350
Total	163 (97.0%)	662 (98.5%)	
Partial	4 (2.4%)	9 (1.3%)	
Dependent	1 (0.6%)	1 (0.1%)	
RAND-36			
Physical functioning	82.33 ± 20.96	87.12 ± 18.37	0.004^*^
Role limitations attributable to physical health	72.22 ± 39.80	82.68 ± 34.78	0.002^*^
Role limitations attributable to emotional problems	76.71 ± 38.75	87.72 ± 30.41	0.001^*^
Energy/fatigue	60.74 ± 20.15	69.13 ± 19.71	<0.001^*^
Emotional well-being	65.57 ± 18.04	73.69 ± 17.50	<0.001^*^
Social functioning	82.45 ± 18.82	90.19 ± 15.15	<0.001^*^
Pain	78.04 ± 19.49	84.06 ± 17.93	<0.001^*^
General health	57.04 ± 21.47	63.77 ± 19.77	<0.001^*^
Sum score of eight domains	580.64 ± 150.13	638.93 ± 114.56	<0.001^*^
Sleep quality			
Poor sleeper (PSQI ≥5)	98 (58.3%)	363 (58.8%)	0.907
PSQI total	6.07 ± 3.87	5.95 ± 3.47	0.721
Sleep quality	1.49 ± 0.86	1.25 ± 0.86	0.001^*^
Sleep latency	1.30 ± 0.97	1.16 ± 0.98	0.096
Sleep efficiency	0.79 ± 1.17	0.49 ± 0.97	0.003^*^
Sleep duration	1.21 ± 1.04	1.08 ± 0.92	0.115
Sleep disturbance	0.05 ± 0.26	1.11 ± 0.65	<0.001^*^
Need medication for sleep	0.58 ± 1.13	0.33 ± 0.86	0.008^*^
Daytime dysfunction	0.64 ± 0.74	0.52 ± 0.70	0.076

PSQI, Pittsburg Sleep Quality Index; TBI, traumatic brain injury.

Sleep quality, assessed using the PSQI, did not vary significantly between the TBI and non-TBI groups (PSQI total score: 6.07 ± 3.87 vs. 5.95 ± 3.47, respectively; *p* = 0.721). However, compared with the non-TBI group, the TBI group had poor sleep quality (1.25 ± 0.86 vs. 1.49 ± 0.86, respectively; *p* = 0.001) and sleep efficiency (0.49 ± 0.97 vs. 0.79 ± 1.17, respectively; *p* = 0.003). Moreover, compared with the non-TBI group, the TBI group exhibited an increased reliance on medication to aid sleep (0.33 ± 0.86 vs. 0.58 ± 1.13, respectively; *p* = 0.008) and alleviate sleep disturbances (1.11 ± 0.65 vs. 0.05 ± 0.26, respectively; *p* < 0.001; Table 6).

### Effects of medication/substance use on the quality of life of patients with traumatic brain injury

**FIG. 2.**
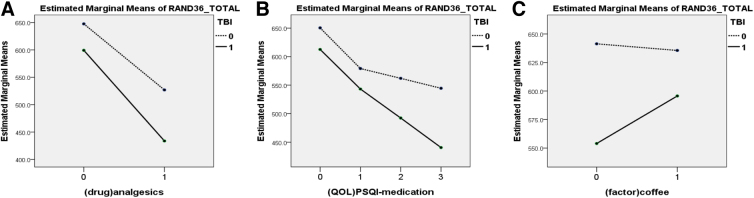
Effects of TBI and medication/substance on health-related quality of life. The use of analgesics and experiencing TBI had a negative impact on the quality of life, as measured by the RAND-36 score (**A**). Additionally, the use of sleep aid medication and being in the TBI group also had a negative impact on quality of life (**B**). However, there was no noticeable interaction between these groups (*p* > 0.05). Further investigation of the interaction between TBI and substance use revealed a significant interaction between TBI/non-TBI groups and coffee consumption/non-consumption on quality of life (**C**). Regular coffee consumption had a more positive effect on quality of life in the TBI group as compared to the non-TBI group (*p* = 0.045). PSQI, Pittsburgh Sleep Quality Index; QOL, quality of life; TBI, traumatic brain injury.

The results of the two-way analysis of variance for two independent groups and their interaction are presented in Figure 2. Quality of life, measured as RAND-36_total, was significantly affected in both patients with a history of TBI (*F* = 15.860; *p* < 0.001) and persons using analgesics (*F* = 64.916; *p* < 0 .001); however, no interaction was noted between the aforementioned two groups (*F* = 1.599; *p* = 0.206; Fig. 2A). Similar results were observed in the relationship between TBI and the need for sleep medication. Both TBI (*F* = 8.596; *p* = 0.003) and sleep medication use (*F* = 26.710; *p* < 0.001) individually affected the RAND-36 sum score, but no significant interaction was observed between the two groups (*F* = 1.353; *p* = 0.256; Fig. 2B). However, a statistically significant interaction was observed between the effects of TBI and coffee use on RAND-36_total (*F* = 4.034; *p* = 0.045; Fig. 2C). Besides, no significant interaction was observed between TBI and cigarette smoking, betel nut chewing habit, regular tea use, or alcohol use (score on the Alcohol Use Disorders Identification Test; all *p* > 0.05).

### Comparison of traumatic brain injury cases reporting with and without initial consciousness impairment

Those who reported experiencing a previous TBI also provided additional details regarding the presence of consciousness impairment at the time of the initial TBI incident. Based on their accounts, we subsequently categorized TBI cases into two groups: those with initial consciousness impairment and those without. Analyses encompassing demographics, outcomes, multi-domain assessments, and substance/drug use revealed no significant group disparities across all comparative facets (Table 7).

**Table 7.** Comparison of TBI Cases Reporting With and Without Initial Consciousness Impairment

	TBI with initial consciousness impairment (*N* = 63)	No initial consciousness impairment (*N* = 105)	*p *value
Demographics			
Age	56.78 ± 12.51	58.90 ± 13.76	0.317
Sex (female)	39 (61.9%)	66 (62.9%)	0.902
Outcomes			
Dependent	1 (1.6%)	4 (4.8%)	0.651
QOL: RAND-36 total score	594.66 ± 146.60	572.73 ± 152.28	0.397
Sleep Quality: PSQI total score	5.63 ± 3.90	6.32 ± 3.85	0.266
Multi-domain assessments			
Risk of sarcopenia: SARC-F score	0.86 ± 1.29	1.10 ± 1.62	0.303
Nutrition: MNA	25.43 ± 3.22	25.07 ± 3.47	0.516
Anxiety: HADS-A	4.39 ± 4.22	5.41 ± 4.18	0.488
Depression: HADS-D	4.44 ± 4.07	4.86 ± 3.63	0.500
Loneliness: ULS-8	15.13 ± 5.23	14.96 ± 5.28	0.844
Subjective cognition: AD8	1.65 ± 2.31	2.21 ± 2.56	0.157
Substance and drug use			
Smoking	23 (36.5%)	34 (32.4%)	0.584
Betel nut chewing	9 (14.3%)	11 (10.5%)	0.460
Coffee	40 (63.5%)	65 (61.9%)	0.837
Tea	31 (49.2%)	52 (49.5%)	0.968
Alcohol: AUDIT score	1.87 ± 2.60	2.68 ± 3.90	0.112
Using analgesics	8 (12.7%)	17 (16.3%)	0.522
Using hypnotics	9 (14.3%)	20 (19.0%)	0.429
PSQI: Need medication for sleep	0.49 ± 1.11	0.64 ± 1.14	0.419

QOL, quality of life; PSQI, Pittsburg Sleep Quality Index; SARC-F, Strength, Assistance with walking, Rise from a chair, Climb stairs, and Falls; MNA, Mini Nutritional Assessment; HADS, Hospital Anxiety and Depression Scale; ULS-8, short-form UCLA Loneliness Scale; AD8, Ascertain Dementia 8; AUDIT, Alcohol Use Disorders Identification Test; TBI, traumatic brain injury.

## Discussion

### Principal findings

We conducted a comparative analysis of community-dwelling persons who had experienced TBI and those who had not. The results revealed that persons with a history of TBI had different lifestyles, including increased rates of analgesic and sleep medicine use; elevated consumption levels of coffee, tea, and betel nut; and increased rates of smoking. Moreover, the TBI group had higher sarcopenia, anxiety, and malnutrition risks as well as poorer sleep quality than did the non-TBI group. Overall, the quality of life of the TBI group was significantly poorer than that of the non-TBI group. These results emphasize the need for multi-domain health promotion strategies aimed at enhancing the overall quality of life of community-dwelling persons recovering from TBI.

### Community-dwelling traumatic brain injury survivors

Community-based studies have predominantly focused on factors that facilitate the reintegration of TBI survivors into their corresponding communities as well as their rehabilitation and home-based care, particularly for patients with severe TBI.^27–30^ However, our TBI community survey revealed that most participants who were able to participate in community cohort recruitment were self-sufficient in their daily lives, with only a minority exhibiting some level of dependence on others. A community integration survey conducted by Lama and colleagues indicated that >95% of all persons who with a history of TBI exhibited independence in community-based activities.^31^ These findings collectively underscore the importance of directing healthcare focus toward mild TBI.

Persons with mild TBI may not immediately seek medical attention because of the absence of severe mobility and cognitive impairments. However, studies on the long-term effects of mild TBI have revealed the potential development of persistent post-concussion syndrome and post-traumatic stress disorder. Patients with mild TBI may experience anxiety, sleep disturbances, and subtle cognitive decline.^32^ Further, their daily lives may be influenced by a reduction in the cognitive processing time of visuospatial and auditory stimuli.^33^ However, attention deficiency and memory complaints should be carefully differentiated from post-traumatic stress disorder, depression, and anxiety in patients with mild TBI.^34^ Therefore, to gain a comprehensive understanding of the requirements of community-dwelling persons who have experienced a TBI, multi-disciplinary approaches, such as those involving extensive neuropsychological evaluations,^34,35^ are essential.

### Quality of life after traumatic brain injury

The effect of TBI on the quality of life is a key concern among TBI survivors. This effect extends across various dimensions, encompassing the physical, emotional, and social aspects of functioning.^36^ The use of assessment tools (e.g., SF-36/RAND-36) helps explore the heterogeneous aspects of TBI-related hazards, and additional TBI-specific instruments may further facilitate the precise measurement of the effect of TBI on patients' quality of life.^37^ For example, an international collaboration created a conceptual model with a specific measure to assess health-related quality of life after TBI. The development of the Quality of Life after Brain Injury (QOLIBRI) initially generated 148 items, and then two multi-center validation studies were conducted to refine the instrument. The final version, called the QOLIBRI, comprises 37 items across six scales related to cognition, self, daily life and autonomy, social relationships, and feeling bothered by emotions and physical problems. The QOLIBRI demonstrated good psychometric properties, including internal consistency (Cronbach's α = 0.75–0.89), test-retest reliability (ranging from 0.78 to 0.85),^38^ and has been validated in the Chinese version for clinicians and researchers related to health-related quality of life after TBI.^39^

Moreover, stratifying TBI survivors by age and sex may offer deeper insights into the effect of TBI on patients' quality of life.^40^ In the pursuit of personalized care for TBI survivors, we should consider several aspects, including the temporal effect (time elapsed post-TBI), age effect (age at the time of TBI and age during healthcare service), injury-related factors (injury mechanism, injury site, injury severity, and brain images), behavioral changes (personality before and after TBI,^41^ stress from TBI, self-coping strategy, health-seeking behavior, and perceived symptoms), and environmental factors (social and physical conditions before and after TBI), which collectively contribute to the diverse clinical outcomes in patients with TBI.^42^ Education regarding acute care, community support, social networking, and family support and their interconnections are crucial for enhancing post-TBI care.^4^

### Changes in the brain after a mild traumatic brain injury

Several mechanisms contribute to a brain injury resulting from trauma. These mechanisms include various processes, such as axonal injury attributable to shearing forces, microglial activation, reactive astrogliosis, and neuroinflammation. These responses manifest through identifiable blood and cerebrospinal fluid biomarkers, including neurofilament, S100 protein, glial fibrillary acidic protein, and ubiquitin carboxyl-terminal hydrolase-L1.^43,44^ In the chronic stage of TBI, the aggregation and deposition of various neurodegenerative proteins, including phosphorylated tau, β-amyloid, α-synuclein, and tar DNA-binding 43 proteins,^45^ may be observed, which accelerates brain atrophy and dementia. Among these proteins, the rapid aggregation and dissemination of tau protein is the most well-known pathology of chronic traumatic encephalopathy.^46,47^ Microvascular injury resulting in the breakdown of the blood–brain barrier may accelerate gliosis and amyloid deposition (in the brain) after a brain trauma.^48^

The brain, an intricately organized and highly differentiated organ, undergoes pathological changes after a TBI; these changes have implications for the intrinsic connectivity of the brain. The changes are dynamic over the post-TBI period and exhibit potential for neuroplasticity. A longitudinal magnetic resonance imaging study of the functional and structural connectivity of the brain after a mild TBI revealed initial functional hypoconnectivity within specific subnetworks of the default mode network as well as initial structural hypoconnectivity in subnetworks associated with central hub areas. These functional and structural hypoconnectivities were anatomically overlapped. Subsequently, the disconnected brain partially recovered, with clinical improvements in memory and attention. However, compensation mechanisms and subtle impairments were observed at the 1-year follow-up.^49^

Additionally, changes in functional connectivity between the default mode network and the frontal lobe were noted on functional magnetic resonance imaging.^50^ Disrupted neural synchrony and impaired network function pertaining to intrinsic and motor dynamics were identified through magnetoencephalography.^51^ Further, the cerebellum, often referred to as the second computational unit of the brain,^52^ exhibited alterations in functional connectivity after a mild TBI.^53,54^

Post-TBI changes in brain connectivity are associated with chronic pain, which explains the increased use of analgesics in our study cohort. Persons experiencing chronic pain after a TBI exhibited changes in functional connectivity between the nucleus accumbens and the primary motor cortex; between the periaqueductal gray matter and the primary somatosensory cortex; and among the periaqueductal gray matter, nucleus accumbens, and rostral anterior cingulate cortex. These brain areas play pivotal roles in the central modulation of pain, stress, anxiety, and reward behaviors.^55–57^

Fatigue and a sense of reduced energy are frequently reported by patients with TBI^58^; these observations are consistent with the findings of our study. The increased fatigability is associated with the changes in functional connectivity among the anterior insula, rostral anterior cingulate cortex, and inferior frontal regions.^59^ Moreover, the reduction in glutamate neurotransmission attributable to astroglia dysfunction may contribute to the development of post-TBI fatigue.^60^ In addition to aerobic exercise and multi-modal care, oral medications such as methylphenidate and melatonin can reduce post-TBI fatigue.^58^ Moreover, increased coffee consumption may be an effective strategy for persons with TBI; this is because caffeine, a central nervous system stimulant, exerts its effects by mobilizing calcium, maintaining cAMP levels by inhibiting phosphodiesterase, and influencing adenosine receptors.^61^ However, in a rat model of TBI, treatment resulted in improved motor function but worsened visuospatial function.^62^ Further research is required to fully understand the benefits and limitations of coffee consumption for patients with TBI.

### Multi-discipline Healthcare for traumatic brain injury survivors

As indicated by our findings, community-dwelling persons with a history of TBI experience higher levels of anxiety, sleep problems, and pain as well as poorer quality of life encompassing various mental and physical aspects than do those without TBI. The higher risk of sarcopenia and lower blood level of vitamin D in the TBI group than in the non-TBI group further suggest potential deficiencies in physical activity and outdoor activities among persons with a history of TBI. Malnutrition may increase the risks of sarcopenia and vitamin D deficiency. To address the multi-faceted health concerns of community-dwelling TBI survivors, we can implement an integrated program aimed at effectively managing and mitigating the long-term health burden associated with mild TBI. A vital component of this approach involves bridging the gap between post-acute care and successful community reintegration. This necessitates a shift in focus from ensuring the regaining of daily function to providing mental support and establishing long-term health support facilities with improved medical accessibility.^4^ The goals of holistic care are restoring functionality and enhancing the quality of life of persons navigating life after a TBI.

Modern cognitive modulation techniques can improve the overall performance of TBI survivors in their work and daily lives, ultimately enhancing their quality of life. A systematic review of neuromodulation treatments for mild TBI revealed that these treatment strategies mitigated symptoms such as post-concussion symptoms, headaches, depression, anxiety, sleep problems, and cognitive function. Further, significant progress was observed in terms of patients' independence, working ability, and overall quality of life. The observed clinical improvements were consistent with electrophysiological features, such as field potentials, hemodynamics features, and electroencephalographic characteristics.^63^ Comprehensive neuromodulation protocols involving whole-brain electroencephalography may help us address a broader spectrum of deficits in patients with TBI. Drawing from our previous experience, the implementation of low-resolution tomography Z-score neurofeedback substantially improved the memory, attention, working productibility, and quality of life of patients with TBI. This technique was superior to the theta/beta neurofeedback approach, which only affected immediate memory and selective attention.^64^

### Limitations

There are several limitations of the study to be mentioned. First, it is important to note that community cohort recruitment was conducted in gathering spots rather than through home visits. This approach may have made it difficult for patients with moderate-to-severe TBI and significant disabilities to participate, resulting in an enrollment restriction to those with mild TBI or relatively better recovery. To overcome this selection bias and cover a complete patient population, future investigations based on the disease cohort are necessary.

Second, although we noticed a higher demand for cigarettes, betel nut, coffee, and tea used in the TBI than non-TBI participants, the study could not provide whether the personal use of these substances were the post-TBI modifications or their original lifestyles; the latter implied a higher incidence of TBI in a specific population. It is conceivable that persons who engaged in substance use or had a pre-existing lower quality of life before the injury might have been more susceptible to experiencing a TBI initially. Further, participants facing challenges related to their quality of life or substance use may more readily recollect their TBI experiences, whereas those without such concerns might not have dwelled on past injuries as extensively. Therefore, the presence of potential recall bias necessitates thorough investigation. It may also be prudent to seek additional clarification through a thorough examination of medical records.

Third, the current study did not provide the underlying brain structural and functional alterations of the increased anxiety, sarcopenia risk, and lifestyle difference. The relationship between brain changes and increased use of analgesics in TBI patients was also warranted for further investigation. To follow-up the community-dwelling TBI patients with neuroimaging studies is a future direction to answer the behavior-brain correlations.

Finally, the inquiries regarding TBI events simply asked for initial consciousness impairment, overlooking other pertinent symptoms such as focal weakness, visual changes, headaches, or seizures. The self-reporting is also susceptible to recall bias, which compromises the precision of the initial severity rating. Further, there was a lack of assistance from a medical record assistant to elucidate the severity of the initial TBI events, potentially resulting in an incomplete representation of TBI severity in the study. This omission could lead to the exclusion of a comprehensive spectrum of TBI severities. These limitations may inadvertently enroll persons with relatively mild TBI, possibly explaining the observed lack of differences in daily life independence between the non-TBI group and TBI subjects, irrespective of the presence or absence of initial consciousness impairment. Addressing these aspects could enhance the comprehensiveness and accuracy of the study's findings.

## Conclusion

In this study, we identified persons with TBI history from the NTCMRC and matched them to non-TBI controls on the basis of age and sex. The TBI and non-TBI groups were similar in terms of demographics, physiological characteristics, and comorbidities. However, the laboratory profiles revealed slight variations between the groups, with the TBI group exhibiting marginally higher blood levels of creatinine and alanine transaminase levels and lower blood levels of bilirubin and vitamin D than did the non-TBI group. A distinctive lifestyle pattern was evident in the TBI group; this pattern was characterized by an increased reliance on analgesics and sleep aids, which negatively affected the quality of life of the TBI group. Moreover, the TBI group consumed more coffee, tea, and betel nuts and exhibited higher smoking rates than did the non-TBI group. Notably, the consumption of coffee improved the quality of life of TBI survivors. The TBI group exhibited higher risks of sarcopenia, malnutrition, and anxiety than did the non-TBI group.

Overall, the TBI group had a reduced health-related quality of life in domains such as physical well-being, mental health, social interactions, pain management, and fatigue. This group also had poorer sleep quality and efficiency than did the non-TBI group. An in-depth exploration of the lives of community-dwelling persons who have experienced TBI revealed significant changes in their lifestyle and quality of life. These unique findings should be hypothesis-generating in prospective studies to reduce recall bias. These findings also emphasize the necessity of comprehensive care for TBI survivors; the care programs should encompass interventions pertaining to mental health support, pain management, nutrition, and exercise.

## Transparency, Rigor, and Reproducibility Summary

The study was pre-registered at ClinicalTrials.gov (identifier: NCT04839796). The analysis plan was not formally pre-registered. A sample size of TBI subjects was planned based on the availability of the community cohort population. Actual sample size was 168 and 672 subjects of TBI and control groups. Data collection was performed by investigators blinded to relevant participant characteristics such as pre-defined group membership. All data sets were analyzed at the same time. Key inclusion criteria and clinical outcomes were assessed by investigators with neurosurgery and neurology professions. Deidentified data from this study are not available in a public archive. Deidentified data from this study will be made available (as allowable according to institutional IRB standards) by e-mailing the corresponding author. The authors agree to publish the manuscript using the Mary Ann Liebert Inc. “Open Access” option under the appropriate license.

## Abbreviations Used

AD8: Ascertain Dementia 8
HADS: Hospital Anxiety and Depression Scale
MetS: metabolic syndrome
MNA: Mini Nutritional Assessment
NTCMRC: Northeastern Taiwan Community Medicine Research Cohort
PSQI: Pittsburgh Sleep Quality Index
QOLIBRI: Quality of Life after Brain Injury
SARC-F: Strength, Assistance in walking, Rise from a chair, Climb stairs, and Falls
TBI: traumatic brain injury
